# Outcomes of Endovascular Aneurysm Repair (EVAR) Compared to Open Repair in Abdominal Aortic Aneurysm: An Umbrella Meta-Analysis

**DOI:** 10.7759/cureus.63183

**Published:** 2024-06-26

**Authors:** Amrita M Cherian, Rakshaya Venu, Pavithra Ishita Raja, Sabanantham Saravanan, Usman Khan, Rahul Kantawala, Soubarno Tasnim, Naveen J Bose, Rajanikant Kumar, Ruchira Clementina, Nagma Sabu, Saifullah Syed, Anjani Mahesh Kumar Cherukuri, Aizaz R Chaudhry, Alisha Lakhani, Avinash Sharma

**Affiliations:** 1 Medicine, Post Graduate Institute of Medical Sciences and Research (PGIMSR) Employees' State Insurance Corporation (ESIC) Medical College, Chennai, IND; 2 Internal Medicine, Saveetha Medical College and Hospital, Chennai, IND; 3 Surgery, Employees' State Insurance Corporation (ESIC) Medical College, Chennai, IND; 4 Medicine, Employees' State Insurance Corporation (ESIC) Medical College, Kolkata, IND; 5 Medicine, Akhtar Saeed Medical and Dental College, Lahore, PAK; 6 Medicine, Smt. N.H.L Municipal Medical College, Ahmedabad, IND; 7 Medicine, Dhaka Medical College , Bangladesh, Dhaka, BGD; 8 Medicine, Madurai Medical College, Madurai, IND; 9 Medicine, Medanta Superspecialty Hospital, Patna, IND; 10 Medicine, Government Medical College, Nizamabad, IND; 11 Medicine, Jonelta Foundation School of Medicine, University of Perpetual Help System DALTA, Las Piñas City, PHL; 12 Medicine, Tallaght Universital Hospital, Dublin, IRL; 13 Medicine, Guntur Medical College, Guntur, IND; 14 Emergency, Bahawal Victoria Hospital, Bahawalpur, PAK; 15 Medicine, Shantabaa Medical College and General Hospital, Amreli, IND

**Keywords:** systematic review and meta-analysis, mortality, evar, endovascular aneurysm repair, abdominal aortic aneurysm

## Abstract

This umbrella meta-analysis aims to investigate two surgical treatments for abdominal aortic aneurysm (AAA): endovascular aneurysm repair (EVAR) and open surgery repair (OSR). Our study aims to elucidate the 30-day mortality rate, reintervention rates, and aneurysm-related mortality in EVAR versus OSR for AAA.

We conducted a comprehensive assessment of meta-analyses (*n* = 34 articles) comparing EVAR and OSR for AAA. We adhered to the Preferred Reporting Items for Systematic Reviews and Meta-Analyses (PRISMA) protocol and considered statistical significance at *P* ≤ 0.05.

For the 30-day mortality rate, a pooled odds ratio (pOR) of 0.59 (95% confidence interval [CI] 0.45-0.77, *P *= 0.0001, and I2 = 98%) indicates that EVAR was associated with a lower risk of mortality compared to OSR. For reintervention rates, a pOR of 1.33 (95% CI = 0.98-1.82, *P* = 0.11, and I2 = 90%). In aneurysm-related mortality, a pOR of 0.78 (95% CI = 0.63-0.97, *P* = 0.03, and I2 = 43%). In postoperative rupture of aneurysm, a pOR of 3.28 (95% CI = 2.16-4.98, *P* < 0.00001, and I2 = 50%). Furthermore, when analyzing systemic complications, only for visceral ischemia, significant results showed lower odds for EVAR, with a pOR of 0.57 (95% CI = 0.40-0.80, *P* = 0.001, and I2 = 0%) was found.

EVAR is better in terms of short-term mortality rate and aneurysm-related mortality. Furthermore, EVAR is still a safer procedure in elective settings, as the studies we included recruited patients for this setting. However, given the high reintervention rates and recent developments in surgical techniques and materials, more recent data and extensive research are needed.

## Introduction and background

Abdominal aortic aneurysms (AAAs) are aortic aneurysms that occur in the infrarenal aorta, the region of the abdominal aorta that is distal to the renal arteries. AAAs should be suspected when the ratio of the infrarenal to undilated suprarenal aorta diameters is more than or equivalent to 1.5. According to the aorta's diameter, AAAs can be divided into two categories: small (not considered for repair, 55 mm) and large (55 mm), when surgical repair can be considered [1]. AAA represents an important public health problem with a prevalence between 1.3% and 12.5%. In women, it generally appears 10 years later than in males. AAA represents about 1% of deaths in males over 65 years old, causing more than 175,000 deaths worldwide. The rupture rates of aneurysms increase markedly with aneurysm diameter; for each 0.5 cm increase in AAA diameter, rates increase by 0.5 mm/year, and rupture rates double [2]. The 13th most common cause of death in the United States is ruptured AAAs [3]. Despite progress in the surgical approach, ruptured AAAs continue to be fatal in most cases, and intraoperative mortality is still significant in ruptured aneurysms [4].

Endovascular aortic repair is a minimally invasive procedure used to treat AAAs, which involves inserting a stent graft into the weakened area of the aorta to reinforce it and prevent rupture. EVAR is often preferred over open surgery due to its lower risk of complications and faster recovery time [5]. Open AAA repair involves creating a larger incision in the abdomen to directly access an aneurysm larger than 5 cm and replacing the weakened portion of the aorta with a synthetic graft. Open surgical repair typically results in higher postoperative complications and longer recovery times compared to EVAR [6].

Evidence shows endovascular repair is associated with lower one-year mortality compared to open surgical repair (2% vs. 14%; *P *= 0.02) [7]. Emergency endovascular repair was associated with a significant reduction in mortality (pooled odds ratio [pOR] 0.624; *P *< 0.0001) and the ICU stay was reduced by four days (pooled effect size estimate -0.70; *P* = 0.0001) [4,8]. The mortality rate for ruptured AAA after the endovascular repair was 24%, which was also lower than open repair of the ruptured AAA [6,9]. Though endovascular repair has many advantages, technical complications such as endoleak, migration, and stent wire fracture can occur requiring reintervention, with reported long-term rates of reintervention as high as 30%. Like open repair, endovascular repair is associated with respiratory, cardiac, renal, neurological, and hemorrhagic complications [10].

Studies have variable sample sizes, study designs, differences in the characteristics of patients in both groups, and different definitions for outcomes, leading to low or moderate heterogeneity of the results. Studies also have a high level of publication bias. This makes us exercise caution while interpreting these results [10,11]. Numerous studies either neglected to report long-term data or showed a large difference in the length of follow-up among the studies analyzed. Estimates of long-term postoperative outcomes and reintervention risk are also limited [12]. Reports are linking secondary rupture after a previous EVAR to greater perioperative mortality; however, there is not enough data to draw firm conclusions.

## Review

Methodology 

We performed an umbrella review of meta-analysis and systematic reviews on the mortality or survival and long-term outcomes of endovascular aneurysm repair (EVAR) compared with open repair in the management of abdominal aortic aneurysms. It was conducted following the Preferred Reporting Items for Systematic Reviews and Meta-Analyses (PRISMA) guidelines [13].

Search strategy 

An extensive search for meta-analysis that examined the mortality outcomes for EVAR with open repair in the management of AAA was carried out in the PubMed database, which described the characteristics of both this treatment from the year 2002 to 2022 and in English literature. The keywords and their synonyms were searched using appropriate truncations, wildcards, and proximity searching. The terms used to search were (Abdominal Aortic Aneurysm OR AAA) AND (Endovascular aneurysm repair OR EVAR OR endovascular OR endovascular aneurysm repair) AND (open repair AND OR) AND (long-term outcomes OR mortality OR in hospital mortality OR early outcome OR late outcome) AND (meta-analysis OR meta-analysis OR systematic review).

Study selection

The initial search was conducted using the titles of the studies after reviewing the abstracts of the selected studies. Each potential anticipated study was further examined by two investigators independently, which matched the predetermined inclusion criteria. Any disagreement was sought through discussion with another investigator. 

Eligibility criteria and data collection

We included all the meta-analyses that had randomized controlled trials and observational studies, which were prospective cohort, retrospective cohort, or case-controlled studies, showing quantitative data on the mortality and long-term complications outcomes. All types of aneurysmal presentation were taken into consideration, and meta-analyses showing data on patients presenting with ruptured AAA or getting an elective treatment were included. Mortality or survival data were selected if the study mentioned it as 30-day mortality, perioperative mortality, short-term mortality, or immediate postoperative mortality. Long-term complications considered included reintervention or reoperation, aneurysm-related mortality, postoperative rupture of an aneurysm, and systemic complications such as cardiac events, myocardial infarction, renal issues, pulmonary complications, cerebrovascular events or stroke, visceral ischemia, limb ischemia, wound infection, amputation, abdominal compartment syndrome, and endoleak. Review articles, animal studies, non-English texts, and meta-analyses with insufficient data were excluded from the study. Meta-analyses with the same topic and outcome were included, and we obtained those pooled estimated effects for our meta-analysis. 

We obtained details of the included studies, study design type, sample size, and data on various measured outcomes, which were entered into Excel for final analysis. The estimated effect from those meta-analyses was considered in the random effect model at a 95% confidence interval (CI). 

Statistical analysis

On selecting the meta-analyses, we found that the individual meta-analysis had studies included from different countries, different ethnic groups, and genders, and also the majority of them had effect size measured in random effect at 95% CI. Hence, we performed the pooled effect estimation from those meta-analyses at 95% CI using random-effects models for generic inverse variance data in RevMan version 5.4. Heterogeneity across the selected studies was assessed using I2 statistics, with I2 > 75% considered indicative of significant heterogeneity among the included studies. *P*-value < 0.05 was considered significant for the overall pooled estimated effect. 

Search strategy for the included studies

Initially, 146 potential studies were obtained by entering the keywords in the selected database, which were sought for screening. Out of that, 8 of the studies were repeated, 21 were excluded from the title itself as it did not match our objectives, and 2 were case reports. Abstracts were then searched for the remaining studies. Out of these, 21 were excluded because they were only systematic reviews without meta-analysis, 11 studies lacked a comparison group, 4 studies examined the influence of gender on mortality regardless of the treatment offered, 41 did not meet the inclusion criteria or the desired outcome, and data could not be retrieved from 4 studies even after contacting the corresponding authors. Finally, 34 studies were included in the meta-analysis.

**Figure 1:**
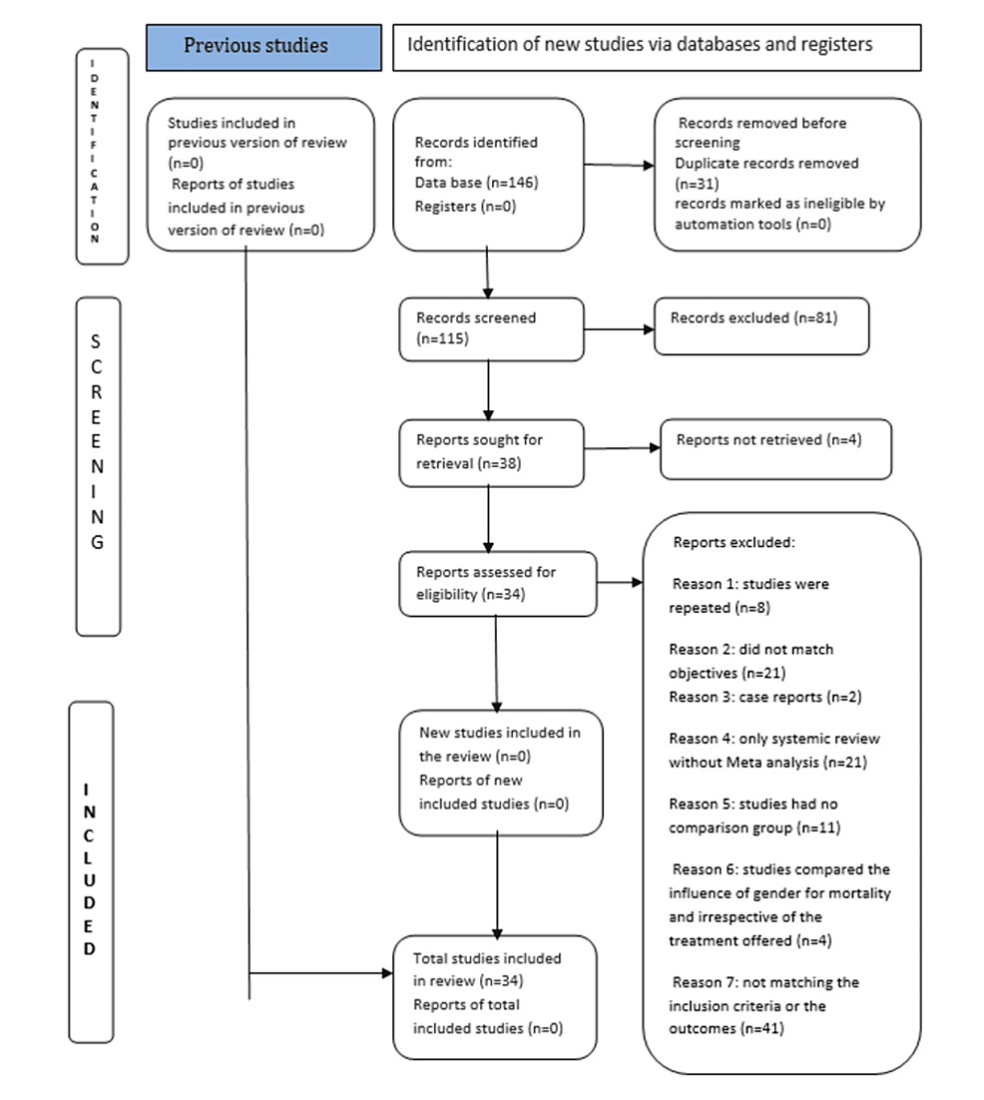
Preferred Reporting Items for Systematic Reviews and Meta-Analyses (PRISMA) diagram.

Overall, 30-day mortality was reported as an outcome in 27 studies, reintervention in 19 studies, aneurysm-related mortality in 10 studies, postoperative rupture in 4 studies, cardiac complications in 5 studies, myocardial infarction in 2 studies, renal complications in 8 studies, pulmonary complications in 4 studies, cerebrovascular complications in 5 studies, visceral ischemia in 4 studies, limb ischemia in 3 studies, amputation as a complication in 1 study, abdominal compartment syndrome in 1 study, and wound infection in 2 studies (Figure 1).

Results

**Figure 2:**
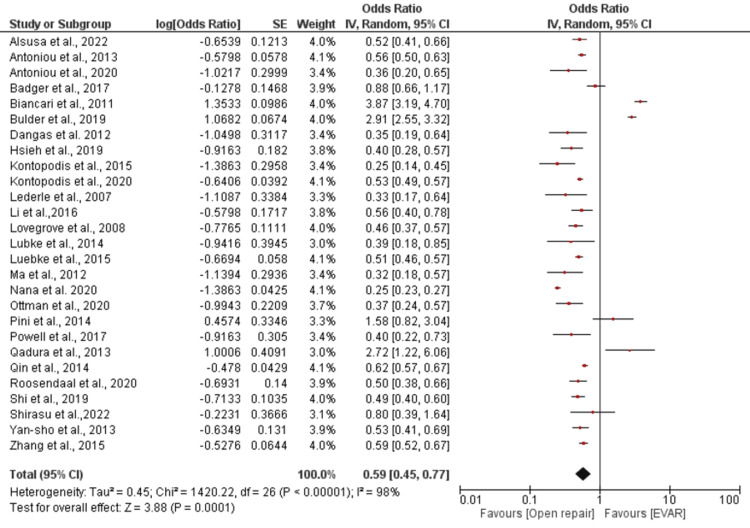
Forest plot showing the pooled effect size from individual meta-analyses for 30-day mortality in EVAR compared to open repair in patients with abdominal aortic aneurysm. [10-12,14-37]. EVAR, endovascular aneurysm repair; SE, standard error; CI, confidence interval

Our meta-analysis compared EVAR and open repair for various primary outcomes related to AAA surgery. We found a statistically significant pOR of 0.59 (95% CI 0.45-0.77; *P* = 0.0001) for 30-day mortality, indicating that EVAR was associated with a lower risk of mortality compared to open repair (Figure 1). There was significant heterogeneity among the included studies, with an I2 of 98% and *P* < 0.00001. We also found that the rates of reintervention, readmission, and reoperation after EVAR and open repair were not significantly different, with a pooled estimated effect of 1.33 and a *P*-value of 0.11 (Figure 2).

Thirty-Day Mortality Outcome in Patients Undergoing EVAR Compared to Open Repair 

When comparing the two treatments, the logarithm of the estimated effect for 30-day mortality, perioperative mortality, or immediate mortality was entered for analysis from individual meta-analyses. This showed an overall pooled estimated effect of 0.59, with a 95% CI of 0.45 to 0.77, and a *P*-value of 0.0001 in a random-effects model (Figure 2).

Reintervention

Reintervention, readmission, or reoperation after EVAR or open repair was found to be not significant, with an overall pooled estimated effect of 1.32, 95% CI of 0.94 to 1.86, and *P*-value = 0.11.

**Figure 3:**
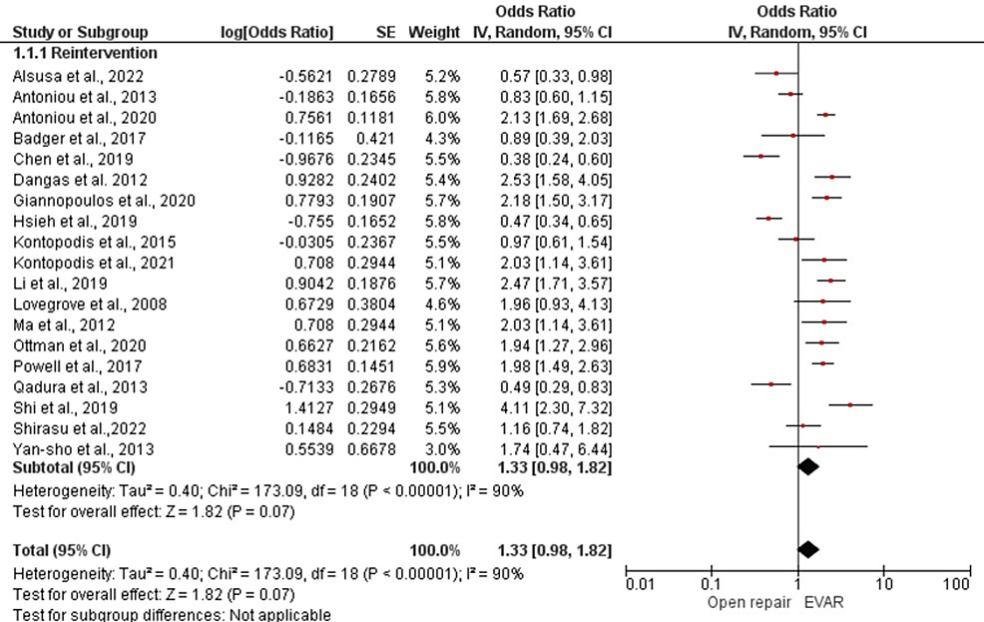
Forest plot for reintervention in EVAR versus open repair in patients with abdominal aortic aneurysm. [10,11,14-17,20,23,26,28,30,31,34-36,38-41]. EVAR, endovascular aneurysm repair; SE, standard error; CI, confidence interval

**Figure 4:**
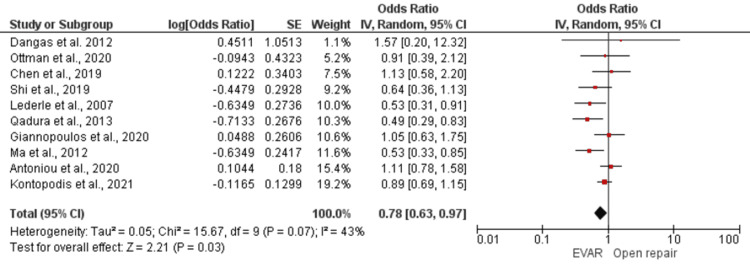
Forest plot for aneurysm-related mortality in EVAR versus open repair in patients with abdominal aortic aneurysm. [16,20,21,26,28,31,34,38,39,40]. EVAR, endovascular aneurysm repair; SE, standard error; CI, confidence interval

In addition, our meta-analysis evaluated the effectiveness of EVAR and open repair in reducing aneurysm-related deaths and the risk of recurrent aneurysm rupture. We found a statistically significant pooled estimated effect of 0.78 (95% CI 0.63-0.97; *P* = 0.03) for aneurysm-related deaths, indicating that EVAR was associated with a lower risk of aneurysm-related deaths compared to open repair (Figure 3). However, we observed moderate heterogeneity among the included studies, with an I2 statistic of less than 75% (*P* = 0.07). On the other hand, the overall effect of recurrent aneurysm rupture was higher in the EVAR-treated group compared to open repair, with an effect estimate of 3.28 (95% CI 2.16-4.98, *P* < 0.00001), which was statistically significant. Heterogeneity among the included studies was moderate, with an I2 statistic of less than 75% (Figure 4).

Aneurysm-Related Mortality 

Compared to open repair, EVAR had lower odds of causing aneurysm-related deaths, with a pooled overall estimated effect of 0.78 at a 95% CI of 0.63 to 0.97 and *P*-value = 0.03, which was statistically significant.

Postoperative Rupture of Aneurysm 

The overall effect of having rupture again of the aneurysm was higher in the EVAR-treated group compared to open repair, with an effect estimate of 3.28, at a 95% CI of 2.16 to 4.98 and a *P*-value < 0.00001, which was statistically significant.

**Figure 5:**
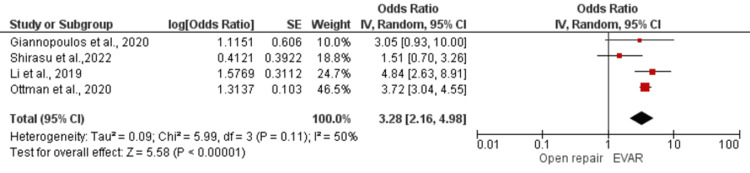
Forest plot for postoperative rupture of an aneurysm in EVAR versus open repair in patients with abdominal aortic aneurysm. [28,35,39,41]. EVAR, endovascular aneurysm repair; SE, standard error; CI, confidence interval

As for our secondary outcome, we found that only for visceral ischemia, EVAR had lower odds compared to open repair, with an overall effect estimation of 0.57 (95% CI 0.40-0.80; *P *= 0.001), which was statistically significant (Figure 5). Heterogeneity among the included studies was found to be I2 = 0%, with *P*-value = 0.48. However, odds for other complications such as cardiac, myocardial, renal, pulmonary, neurological, endoleak, infections, abdominal compartment syndrome, and amputation individually, as well as overall odds of having any systemic complication, were not found to be significantly different between EVAR and open repair.

Systemic Complications

**Figure 6:**
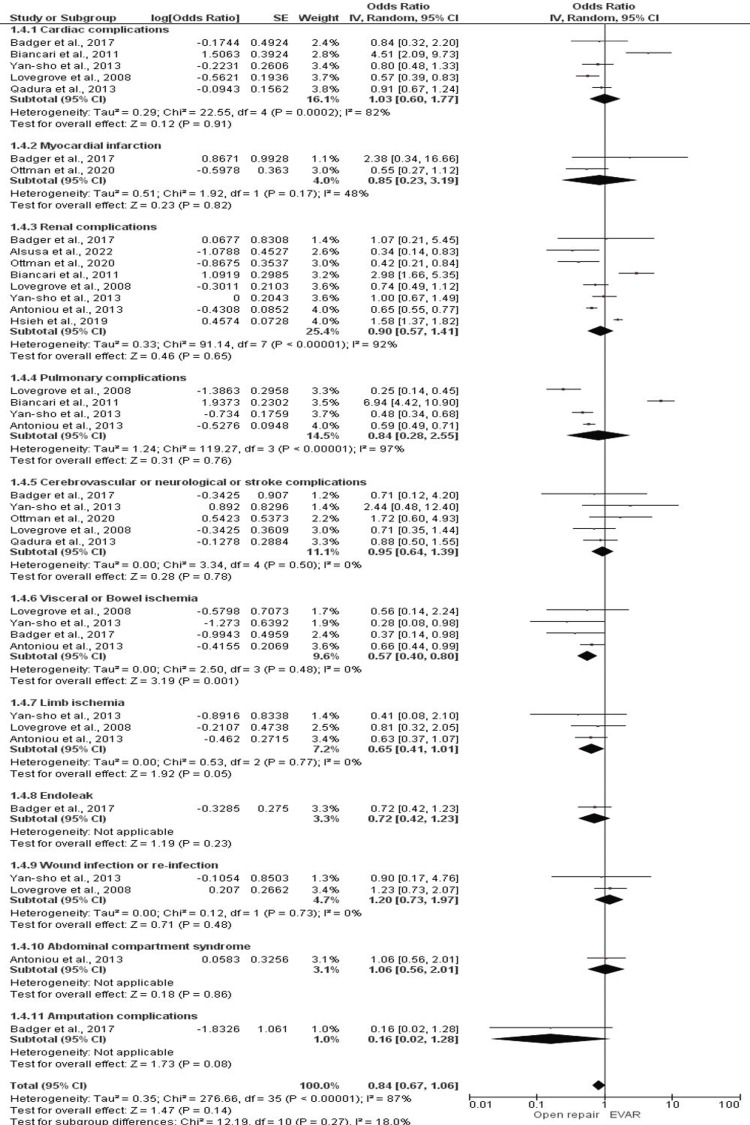
Forest plot for systemic complications in EVAR versus open repair in patients with abdominal aortic aneurysm. Cardiac complications: [17,18,23,31,36]. Myocardial infarction: [17,28]. Renal complications: [11,14,15,17,18,23,28,36]. Pulmonary complications: [15,18,23,36]. Cerebrovascular or neurological or stroke complications: [17,23,28,31,36]. Visceral or bowel ischemia: [15,17,23,36]. Limb ischemia: [15,23,36] Endoleak: [17]. Wound infection or re-infection: [23,36]. Abdominal compartment syndrome: [15]. Amputation complications: [17]. EVAR, endovascular aneurysm repair; SE, standard error; CI, confidence interval

Only for visceral ischemia, EVAR had lower odds compared to open repair, with an overall effect estimation of 0.57, at a 95% CI of 0.40 to 0.80, with a *P*-value = 0.001, which was statistically significant, I2 = 0%, and *P*-value for I2 = 0.48. Odds for other complications individually and overall odds of having any systemic complication were not statistically significant (Figure 6).

Discussion 

The umbrella meta-analysis found that EVAR has a lower 30-day mortality rate compared to open repair in patients with AAAs, with a 41% reduction in mortality (*P*-value = 0.0001). There is no significant difference in reintervention rates, readmission, or reoperation between EVAR and open repair. EVAR is associated with a lower risk of aneurysm-related deaths, and the risk of postoperative rupture is higher with EVAR than open repair. EVAR has lower odds of causing visceral ischemia, but no significant difference in other systemic complications. Clinicians and patients can use these findings to make informed decisions regarding the potential benefits and risks of EVAR versus open repair for AAAs.

Patients treated with EVAR had a lower probability of 30-day mortality compared to open repair. Our findings were similar to other studies, which showed a relative risk (RR) of 0.50 and *P *< 0.001 [18]. Elective EVAR was associated with lower immediate postoperative mortality and morbidity risk than open repair in patients [30]. However, we could not identify this due to lack of studies. Studies have shown that the early survival of EVAR was lost after six months. The early survival advantage of EVAR was due to lower 30-day operative mortality compared to open repair, but this advantage was lost after six months [26]. We found that the odds of reintervention were higher in EVAR (OR 1.33), but the results did not reach significance (CI 0.98-1.821). 

We found that the risk of aneurysm-related mortality is lower with EVAR compared to open repair (OR 0.78, CI 0.63-0.971), which aligns with the findings of Ma et al. [26,31,41]. Another study showed there was no difference in long-term all-cause mortality between the two groups (RR, 0.97; 95% CI 0.86-1.10) [30]. A common complication after EVAR was type II endoleak seen in 11.7%, necessitating reintervention [17]. We believe that since the risk of reintervention is higher in EVAR, the possibility of aneurysm-related mortality can go up during the reintervention procedure, leading to higher long-term mortality [28]. The lifetime need for reintervention after EVAR repair approaches 20%. Reintervention is needed in graft rupture, endoleak, para-anastomotic aneurysm, graft replacement, and graft occlusions/stenoses [31]. There could also be a preoperative selection bias in EVAR that accounts for reduced early mortality rates. The time elapsed for diagnosis from hospital admission to obtaining CT angiography and endovascular planning is often an overlooked element in EVAR. Studies should look into such factors when estimating perioperative mortality rates [29]. In terms of other outcomes, we found that only for visceral ischemia, EVAR had lower odds compared to open repair (OR 0.57, 95% CI 0.40-0.80; *P *= 0.001), and all other systemic complications did not achieve significance. Shirasu et al. also reported similar results, finding no statistically significant differences between the two groups in terms of recurrent infection-related rupture or death or perioperative death [35].

Our study had several limitations. We were unable to address age-related differences or specific rates associated with elective and emergency repairs. Additionally, we could not identify details such as the population studied, selection of centers, adherence to national standards of care, or the length of hospital stay. Furthermore, the studies included in our analysis recruited patients several years ago, and newer devices introduced since then were not covered in this study. Future research should incorporate recent advancements in the field.

## Conclusions

Our meta-analysis comparing EVAR and open surgery repair (OSR) as surgical treatments for AAA revealed several important findings. EVAR demonstrated a significant reduction in 30-day mortality compared to open repair, which highlighted the significance of EVAR in improving survival outcomes.

EVAR is better than open repair in terms of 30-day mortality rate and aneurysm-related mortality. Furthermore, EVAR is still a safer procedure, especially in elective settings, as the studies we included recruited patients from this setting. However, given the high reintervention rates and recent developments in surgical techniques and materials, more recent data and extensive research are needed.
